# Merkel Cell Polyomavirus Small T Antigen Mediates Microtubule Destabilization To Promote Cell Motility and Migration

**DOI:** 10.1128/JVI.02317-14

**Published:** 2014-12-16

**Authors:** Laura M. Knight, Gabriele Stakaityte, Jennifer, J. Wood, Hussein Abdul-Sada, David A. Griffiths, Gareth J. Howell, Rachel Wheat, G. Eric Blair, Neil M. Steven, Andrew Macdonald, David J. Blackbourn, Adrian Whitehouse

**Affiliations:** a School of Molecular and Cellular Biology, University of Leeds, Leeds, United Kingdom; b Astbury Centre for Structural Molecular Biology, University of Leeds, Leeds, United Kingdom; c School of Cancer Sciences, College of Medical and Dental Sciences, University of Birmingham, Birmingham, United Kingdom; d School of Biosciences & Medicine, University of Surrey, Surrey, United Kingdom

## Abstract

Merkel cell carcinoma (MCC) is an aggressive skin cancer of neuroendocrine origin with a high propensity for recurrence and metastasis. Merkel cell polyomavirus (MCPyV) causes the majority of MCC cases due to the expression of the MCPyV small and large tumor antigens (ST and LT, respectively). Although a number of molecular mechanisms have been attributed to MCPyV tumor antigen-mediated cellular transformation or replication, to date, no studies have investigated any potential link between MCPyV T antigen expression and the highly metastatic nature of MCC. Here we use a quantitative proteomic approach to show that MCPyV ST promotes differential expression of cellular proteins implicated in microtubule-associated cytoskeletal organization and dynamics. Intriguingly, we demonstrate that MCPyV ST expression promotes microtubule destabilization, leading to a motile and migratory phenotype. We further highlight the essential role of the microtubule-associated protein stathmin in MCPyV ST-mediated microtubule destabilization and cell motility and implicate the cellular phosphatase catalytic subunit protein phosphatase 4C (PP4C) in the regulation of this process. These findings suggest a possible molecular mechanism for the highly metastatic phenotype associated with MCC.

**IMPORTANCE** Merkel cell polyomavirus (MCPyV) causes the majority of cases of Merkel cell carcinoma (MCC), an aggressive skin cancer with a high metastatic potential. However, the molecular mechanisms leading to virally induced cancer development have yet to be fully elucidated. In particular, no studies have investigated any potential link between the virus and the highly metastatic nature of MCC. We demonstrate that the MCPyV small tumor antigen (ST) promotes the destabilization of the host cell microtubule network, which leads to a more motile and migratory cell phenotype. We further show that MCPyV ST induces this process by regulating the phosphorylation status of the cellular microtubule-associated protein stathmin by its known association with the cellular phosphatase catalytic subunit PP4C. These findings highlight stathmin as a possible biomarker of MCC and as a target for novel antitumoral therapies.

## INTRODUCTION

Merkel cell carcinoma (MCC) is an aggressive skin tumor (1). The reported cases of MCC have tripled in the past 20 years in both Europe and the United States (2), due to an increase in known risk factors—UV exposure, immune suppression, and increased age (1, 3). The cancer is characterized by significant incidence of local recurrence, early involvement of local lymph nodes, and distant metastasis (4). As such, MCC has a poor 5-year survival rate, due to its high propensity to metastasize (5).

Merkel cell polyomavirus (MCPyV) is clonally integrated in ∼80% of MCC tumors (6). MCPyV encodes both large and small T antigens (LT and ST, respectively), which are regulatory proteins required for viral replication and tumorigenesis (6). MCPyV infection and integration occur prior to expansion and metastasis of the tumor (7, 8), and truncation mutations of the LT gene are observed in the integrated genome rendering the virus replication defective (6). LT and ST are required for MCC cell survival and proliferation, as depletion of these T antigens leads to cell arrest and death of MCPyV-positive MCC cells (9). In contrast to simian virus 40 (SV40), MCPyV ST is sufficient to transform rodent cells to anchorage- and contact-independent growth and also induces serum-free proliferation of human cells (10). However, the exact contribution of ST to MCC cell growth is under debate as several ST depletion studies have shown differential dependence for MCC proliferation (11, 12). Recent analyses suggest that MCPyV ST is multifunctional in nature (13). MCPyV ST leads to the hyperphosphorylation of 4E-BP1, resulting in the deregulation of cap-dependent translation, (10), it targets the cellular ubiquitin ligase SCF^Fwb7^, stabilizing MCPyV LT and several cellular oncoproteins (14), and also functions as an inhibitor of NF-κB-mediated transcription (15). Although these interactions are attributed to either MCPyV ST-mediated cellular transformation or MCPyV replication processes, to date, no studies have investigated any potential link between MCPyV T antigen expression and the highly metastatic nature of MCC. This is of significant importance as dissemination and metastasis correlate with poor MCC survival rates (16).

Despite the clinical importance, the molecular basis by which cancer cells acquire the capability to migrate from the primary tumor remains to be fully elucidated (17). What is clear is that cell motility, migration, and invasion are critical factors regulating dissemination (18, 19). The importance of the actin cytoskeletal network in cell motility and migration has since been established (20), and in recent years, it has become evident that the microtubule network also has an important role in facilitating cell motility and migration (21, 22).

Microtubules are essential components of the cytoskeleton and play a crucial role in a variety of cellular functions from the positioning of organelles, providing tracts for long distance transport, to ensuring the correct and timely segregation of chromosomes during mitosis (23–26). Furthermore, the microtubule network is central in controlling cell shape and polarized cell motility (21, 22). Microtubules are composed of αβ-tubulin heterodimers, which self-assemble and disassemble, allowing transitions between microtubule growth and microtubule shrinkage or catastrophe (27, 28). This dynamic process is regulated by microtubule-stabilizing and -destabilizing proteins (23, 29). One such microtubule-associated protein (MAP) is stathmin, also known as oncoprotein-18 (30–32). In its unphosphorylated form, stathmin binds and sequesters tubulin dimers, forming a T_2_S complex and thereby reducing the substrate for growing microtubule polymers, thus indirectly leading to microtubule destabilization. In contrast, phosphorylation of stathmin at one or more serine residues weakens the tubulin-binding ability of stathmin and increases free tubulin concentrations for microtubule assembly (33, 34).

Stathmin overexpression is a feature of multiple cancer types and correlates with poor prognosis and high metastatic potential (35, 36). As such, alterations in the microtubule network by stathmin deregulation play a crucial role in cell motility, migration, and invasion. This is supported by RNA interference (RNAi) studies showing that a reduction in stathmin correlates with reduced cell migration in various cancers (37–40). Moreover, regulation of the phosphorylation status of stathmin can affect its microtubule-destabilizing activity and transforming potential (41, 42). Together these observations highlight an important link between stathmin, microtubule dynamics, and cell motility in cancer.

Herein we utilized quantitative proteomics to examine the effect of MCPyV ST on the host cell proteome. Intriguingly MCPyV ST promotes differential expression of cellular proteins implicated in microtubule-associated cytoskeletal organization and dynamics, leading to a motile and migratory phenotype. Specifically, we demonstrate that stathmin is required for MCPyV ST-mediated microtubule destabilization and cell motility. Moreover, regulation of this process involves the cellular phosphatase catalytic subunit protein phosphatase 4C (PP4C).

## MATERIALS AND METHODS

### Plasmids, siRNAs, and antibodies.

Expression vectors for enhanced green fluorescent protein (EGFP)-ST, EGFP-R7A, and EGFP-STΔ95-111 have been previously described (15). Wild-type EE-PP2A (protein phosphatase 2A), FLAG-PP4C, and transdominant mutants PP2AcH118N and RL-PP4C-HA (hemagglutinin) were kindly provided by Stefan Stack, Marilyn Goudrealt, Brian Hemmings and Tse-Hua Tan, respectively. Stathmin-specific small interfering RNAs (siRNAs) were purchased from Qiagen. Lentivirus-based vectors expressing short hairpin RNAs (shRNAs) targeting T antigens were kindly provided by Masahiro Shuda and Pat Moore (10, 11). Antibodies against stathmin, STRAP, β-tubulin, lamin B, Glu-Glu, Kif14, GAPDH (glyceryaldehyde-3-phosphate dehydrogenase) (Abcam), phosphorylated stathmin, acetylated tubulin (Cell Signaling), dynamin, TBCC (Genetex), FLAG, β-actin (Sigma-Aldrich), and GFP (Living Colors) were purchased from their respective suppliers. The 2T2 antibody was kindly provided by Christopher Buck. Western blot analysis was carried out using specific antibodies at dilutions of 1:1,000 and 1:250, as previously described (43, 44).

### Cells.

i293-ST cells, previously described (15), were maintained in Dulbecco's modified Eagle's medium (DMEM) containing 10% fetal bovine serum (FBS) and 1% penicillin–streptomycin (45, 46). MCC13 and MKL-1 cell lines were maintained in RPMI 1640 medium supplemented with 10% FBS and 1% penicillin–streptomycin. ST-FLAG expression was induced from i293-ST with 2 μg/ml doxycycline hyclate for up to 48 h (15).

### SILAC-based quantitative proteomics.

The protocol for stable isotope labeling by amino acids in cell culture (SILAC) was performed as previously described (47, 48). Essentially i293-ST cells in each 5- by 175-cm^2^ flask containing R6K4 (uninduced) or R0K0 (induced with doxycycline hyclate) were washed and incubated in buffer A for 15 min. The cytoplasm was ruptured using a prechilled Dounce homogenizer. Nuclear pellets were washed in sucrose solution. The R0K0- and R6K4-treated samples were combined for each cellular compartment in a 1:1 ratio, and proteins were separated by SDS-PAGE. Ten gel slices per fraction were extracted and subjected to in-gel digestion using trypsin. Purified peptides were identified using an LTQ-Orbitrap Velos mass spectrometer (University of Bristol Proteomics Facility). Peptide identification and quantification were performed using MaxQuant (49), based on the two-dimensional (2D) centroid of the isotope clusters within each SILAC pair. The derived peak list was searched with the Mascot search engine (version 2.1.04; Matrix Science, London, United Kingdom) against a concatenated database combining 80,412 proteins from the International Protein Index human protein database version 3.6. For quantitative analysis, a 2.0-fold cutoff was chosen as a basis for investigating potential proteome changes (50), using the Database for Annotation, Visualization and Integrated Discovery (DAVID) v6.7 (51).

### Immunoblotting.

Cells were lysed in radioimmunoprecipitation assay (RIPA) buffer supplemented with protease inhibitor cocktail (Roche) (52). Proteins were separated by SDS-PAGE before transfer onto nitrocellulose membrane (Hybond C Extra; Amersham Biosciences). Membranes were probed with the appropriate primary and horseradish peroxidase (HRP)-conjugated secondary antibodies. Proteins were detected using EZ-ECL enhancer solution (Geneflow) as previously described (53).

### Live cell imaging.

Cell motility was analyzed using an Incucyte kinetic live cell imaging system as directed by the manufacturer. A 96-well plate was seeded with cells at a density of 3,000 cells per well. After 12 h, the cells were transfected with 250 ng of DNA/well. Twelve hours later, the transfection medium was changed for 10% Hams F-12 medium, and imaging was started. Imaging was performed for a 24-h period, with images taken every 30 min. Cell motility was then tracked using Image J software (54).

### Multicolour immunohistochemistry.

Formalin-fixed, paraffin-embedded (FFPE) sections from primary MCC tumors were prepared and analyzed as previously described (55). The primary antibodies were cytokeratin 20 (CK20) antibody (dilution 1:50 [Dako]), MCPyV LT antibody CM2B4 (dilution 1:125 [Santa Cruz Biotechnology]), and anti-stathmin 1 antibody (dilution 1:250 [Abcam]). An isotype-matched irrelevant antibody was used as a negative control on serial sections of tissues in parallel, Dako X0943 was used for the CK20 primary antibody, and the rabbit polyclonal isotype control antibody (Abcam) was used to match the stathmin primary antibody. Sections were incubated with appropriate secondary antibodies labeled with different fluorochromes: Alexa Fluor 488 IgG2B and Alexa Fluor 633 IgG2A [Invitrogen] and IgG (H+L)-tetramethyl rhodamine isocyanate (TRITC) (Jackson ImmunoResearch). Nuclear counterstaining was with bis-benzimide (Invitrogen). All slides were mounted with Immu-Mount, and images were captured with a Zeiss LSM 510 confocal microscope (56).

### Scratch wound-healing assay.

Poly-l-lysine-coated 6-well plates were seeded with cells, and the cells were allowed to adhere for 24 h. The cells were then subjected to a scratch using a p1000 pipette tip in a continuous straight line through the well. Images were taken under a Zeiss light microscope at a ×4 magnification at 0, 24, 48, and 72 h postscratch (57). Paclitaxel-based scratch assays were incubated for 4 h with 10 μM paclitaxel after induction for 24 h. siRNA and expression plasmids were transfected 24 h prior to the scratch.

### Invasion and migration assays.

BD BioCoat angiogenesis system assay kits (an endothelial cell migration kit coated with human fibronectin and endothelial cell invasion kit coated with Matrigel) were used as described by the manufacturer's protocol. All conditions were the same for assays performed in triplicate.

### Immunofluorescence.

Immunofluorescence was carried out as previously described. Cells were viewed on a Zeiss 510 confocal microscope under an oil immersion 63× objective lens. Images were analyzed using the LSM imaging software and are presented as z-stacked images (58).

## RESULTS

### MCPyV ST affects microtubule network regulatory proteins.

**FIG 1:**
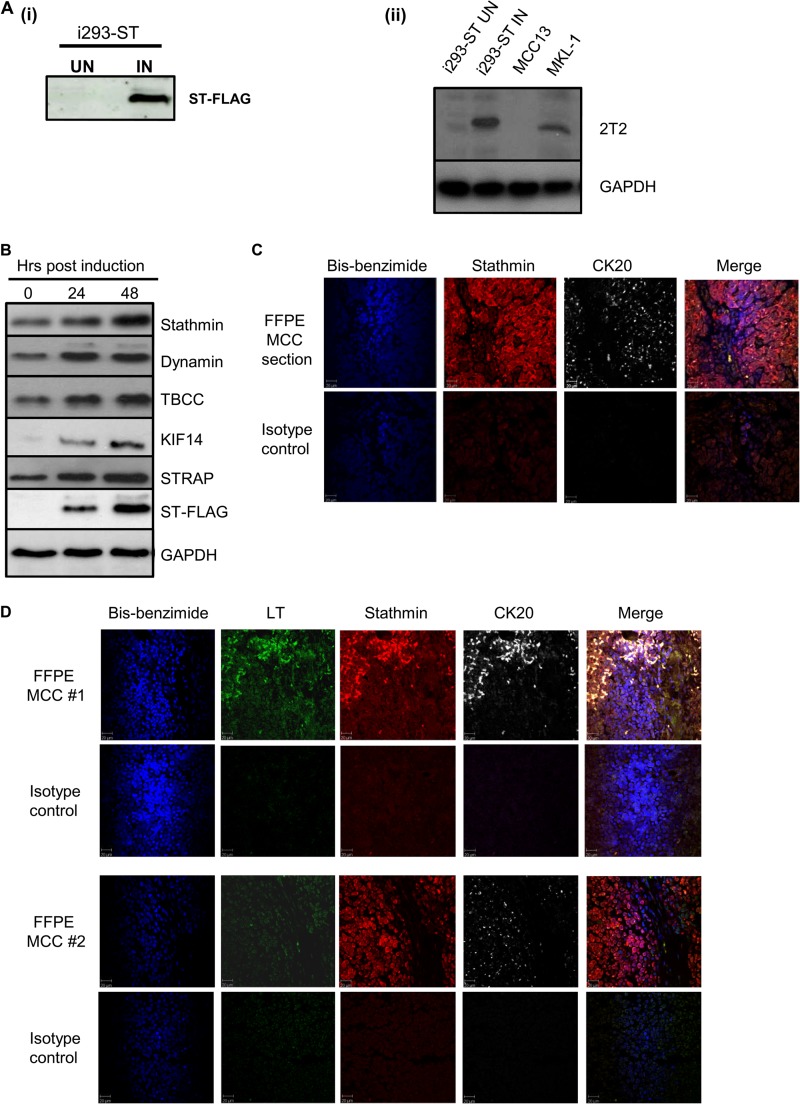
MCPyV ST expression leads to the differential expression of proteins involved in microtubule-associated cytoskeletal organization and dynamics. (A) (i) i293-ST cells were grown in DMEM with R0K0 and induced (IN) for 24 h or grown in DMEM with R6K4 and remained uninduced (UN). Cell lysates were analyzed by immunoblotting with a FLAG-specific antibody. (ii) To confirm that induced levels of MCPyV ST in i293-ST cells are representative of ST expression in the MCPyV-positive MCC cell lines, immunoblotting was performed using an MCPyV T-specific antibody comparing cell lysates from 1 × 10^5^ cells of uninduced and induced i293-ST, MCC13, and MKL-1 cells. (B) i293-ST cells remained uninduced or were incubated for either 24 or 48 h in the presence of doxycycline hyclate. After induction, cell lysates were analyzed by immunoblotting using a FLAG-specific antibody and a range of microtubule-associated-specific antibodies highlighted by quantitative proteomic analysis. GAPDH was used as a measure of equal loading. (C) FFPE sections of a primary MCC tumor were stained with stathmin- and CK20-specific antibodies or an isotype negative control. After washing, sections were incubated with Alexa Fluor-labeled secondary antibodies. Nuclear staining was performed with bis-benzimide. Slides were then analyzed using a Zeiss LSM 510 confocal laser scanning microscope. (D) FFPE sections of two additional primary MCC tumors were stained with stathmin-, MCPyV LT-, and CK20-specific antibodies or an isotype negative control. After washing, sections were incubated with Alexa Fluor-labeled secondary antibodies. Nuclear staining was labeled using bis-benzimide. Slides were then analyzed using a Zeiss LSM 510 confocal laser scanning microscope.

To analyze the effect of MCPyV ST expression on the cellular proteome, SILAC-based quantitative proteomics was performed utilizing a 293 Flp-In cell line capable of inducible MCPyV ST expression, termed i293-ST (15). The i293-ST line is a robust model for this analysis, as inducible levels of MCPyV ST are representative of ST expression in an MCPyV-positive MCC cell line, MKL-1 (Fig. 1A). Bioinformatic analysis using the Database for Annotation, Visualization and Integrated Discovery (DAVID) v6.7 (51) highlighted that a significant proportion of the highly differentially expressed proteins were implicated in gene ontology groupings involving microtubule-associated cytoskeletal organization and dynamics (see Fig. S1 in the supplemental material), which is the focus of this study. Figure S2 in the supplemental material summarizes the differential expression of proteins associated with other gene ontology groupings.

Of particular interest was the dramatic upregulation (8.33-fold) of the microtubule-associated protein stathmin, a critical factor for microtubule dynamics (30). Stathmin is overexpressed in many human cancers and is implicated in clinical characteristics such as tumor grade, size, and prognosis (37). To confirm that stathmin and a selection of other proteins associated with microtubule dynamics were altered upon MCPyV ST expression, immunoblotting was performed comparing uninduced and induced i293-ST cell lysates (Fig. 1B). The results show an increase in the levels of expression of the microtubule-associated proteins upon MCPyV ST expression and support the quantitative proteomic analysis.

To further investigate the differential expression of stathmin with regard to MCC, multicolor immunohistochemistry analysis was performed on formalin-fixed, paraffin-embedded (FFPE) sections of primary MCC tumors. Sections were incubated with cytokeratin 20 (CK20) (a marker widely used to distinguish MCC) and stathmin-specific antibodies. An isotype-matched control was also used as a negative control. The results show demonstrably higher levels of stathmin expression coincident with CK20 staining in regions of the tumor (Fig. 1C). These data suggest that MCC tumor cells express increased levels of stathmin. In addition, multicolor immunohistochemistry analysis was performed on FFPE sections of two further MCC tumors to determine the potential correlation between stathmin expression levels and MCPyV positivity (Fig. 1D). Sections were stained as described above but were also incubated with an MCPyV LT-specific antibody. The results again show abundant levels of stathmin expression coincident with CK20 staining in both MCC tumors; however, interestingly, LT expression was much weaker in one of the tumors, suggesting that other mechanisms of stathmin upregulation may occur in MCC, including possible virus-independent mechanisms.

### MCPyV ST promotes cell motility, migration, and invasion.

**FIG 2:**
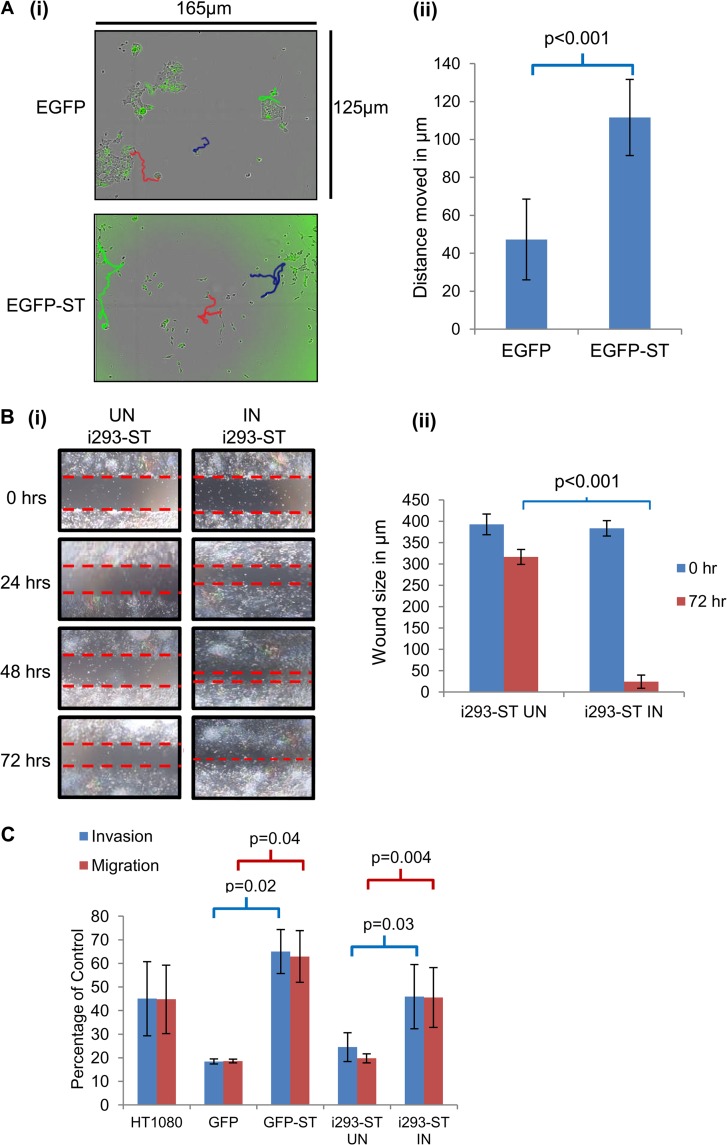
MCPyV ST promotes cell motility, migration, and invasion. (A) (i) HEK293 cells were transiently transfected with either EGFP or EGFP-ST. After 12 h, cell motility was analyzed using an Incucyte kinetic live cell imaging system. Images were taken every 30 min for a 24-h period. The movement of cells was then tracked using Image J software: three cell motility traces for each transfection are shown in each image, colored red, blue, and green. (ii) The average distance moved by pEGFP- or pEGFP-ST-transfected cells was measured in μm (*n* = 50). (B) (i) Poly-l-lysine-coated 6-well plates were seeded with i293-ST cells, and the cells remained uninduced (UN) or were induced (IN) by incubation in the presence of doxycycline hyclate. After 24 h, a scratch was created by scraping the monolayer using a p1000 pipette tip. Cells were then incubated for a 72-h period in the absence or presence of doxycycline hyclate. Migration of cells toward the scratch was observed over a 72-h period, and images were taken every 24 h under a Zeiss light microscope at ×4 magnification. (ii) The size of the wound was measured at 0 and 72 h. Scratch assays were performed in triplicate. (C) Precoated Matrigel and fibronectin-based Transwell migration and invasion tissue culture plates were seeded with uninduced or induced i293-ST cells and EGFP- versus EGFP-ST-transfected cells. HT1080 cells, an invasive human fibrosarcoma cell line, were also used as a positive control. Cells were incubated in the Transwell plates for 22 h and then labeled with calcein AM fluorescent dye for 90 min, and fluorescence was measured. The experiment was performed in triplicate, and the graph indicates the fluorescence (percentage) of cells moving through the migration and invasion plates relative to the uncoated plate, set at 100%.

To assess whether MCPyV ST promotes a more motile and migratory cell phenotype due to the differential expression of microtubule-associated proteins, live cell imaging was performed using an Incucyte kinetic live cell imaging system comparing EGFP- with EGFP-ST-expressing 293 cells. We have previously shown that the EGFP fusion does not affect ST functioning (15). Cells were imaged every 30 min over a 24-h period, and cell motility was tracked using Image J software (Fig. 2A; see Movies S1 and S2 in the supplemental material). The results show line traces following the cell motility of 3 distinct cells, demonstrating that MCPyV ST leads to a statistically significant increase in cell motility. Moreover, imaging demonstrates that in contrast to EGFP-expressing cells, where cells grow in a colony, MCPyV ST-expressing cells rapidly dissociate from the colonies (as highlighted in the top right section of the movies in the supplemental material), suggesting a more motile and migratory phenotype. To confirm this observation, a scratch assay was performed comparing uninduced versus induced i293-ST cells. Cellular growth back was recorded every 24 h for a period of 3 days, and the results indicate that, in contrast to uninduced cells, MCPyV ST enhanced the motility and migration of cells, fully closing the wound at 72 h (Fig. 2B). To verify this enhanced motile and migratory phenotype, Matrigel and fibronectin-based Transwell migration and invasion assays were performed. HT1080 cells, an invasive human fibrosarcoma cell line, were used as a positive control. The results demonstrate that both induced i293-ST and EGFP-ST-expressing cells showed a significant increase in migration and invasive properties compared to uninduced or EGFP-expressing cells, respectively (Fig. 2C).

**FIG 3:**
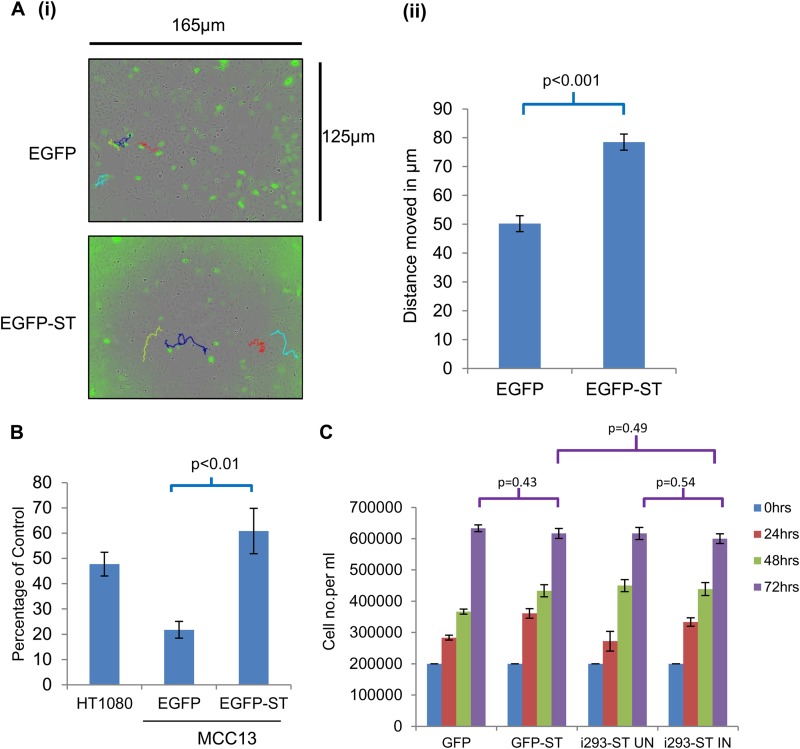
MCPyV ST promotes cell motility and migration in MCC13 cells. (A) (i) MCC13 cells were transiently transfected with either EGFP or EGFP-ST expression vectors. After 12 h, cell motility was analyzed using an Incucyte kinetic live cell imaging system. Images were taken every 30 min for a 24-h period. The movement of cells was then tracked using Image J software: four cell motility traces for each transfection are shown in each image, colored red, blue, cyan, and yellow. (ii) The average distance moved by EGFP- or EGFP-ST-transfected cells was measured in μm (*n* = 50). (B) Precoated Matrigel-based Transwell migration tissue culture plates were seeded with EGFP- versus EGFP-ST-transfected MCC13 cells. HT1080 cells, an invasive human fibrosarcoma cell line, were also used as a positive control. Cells were incubated in the Transwell plates for 22 h and then labeled with calcein AM fluorescent dye for 90 min, and fluorescence was measured. The experiment was performed in triplicate, and the graph indicates the fluorescence (percentage) of cells moving through the migration plates relative to the uncoated plate, set at 100%. (C) A 6-well plate was seeded with 1 × 10^4^ uninduced or induced i293-ST cells and EGFP- versus EGFP-ST-transfected cells in DMEM–10% FCS, and the number of cells was counted every 24 h for a period of 3 days (*n* = 3).

Furthermore, to confirm these observations in an MCC cell line, live cell imaging and Matrigel-based migration assays were repeated using the MCPyV-negative cell line MCC13, transfected with either EGFP or EGFP-ST expression constructs. Similar findings were observed in MCC13 cells, where MCPyV ST increased their cell motility (Fig. 3A) and migration (Fig. 3B). To confirm that these observations were due to enhanced cell motility, migration, and invasion and not to increased cell proliferation, assays were performed in 10% serum levels, the same serum levels used for all motility assays. The results show no statistical difference in cell proliferation upon MCPyV ST expression (Fig. 3C). Together these data suggest that MCPyV ST leads to enhanced cell motility and migration.

### MCPyV ST promotes microtubule destabilization.

**FIG 4:**
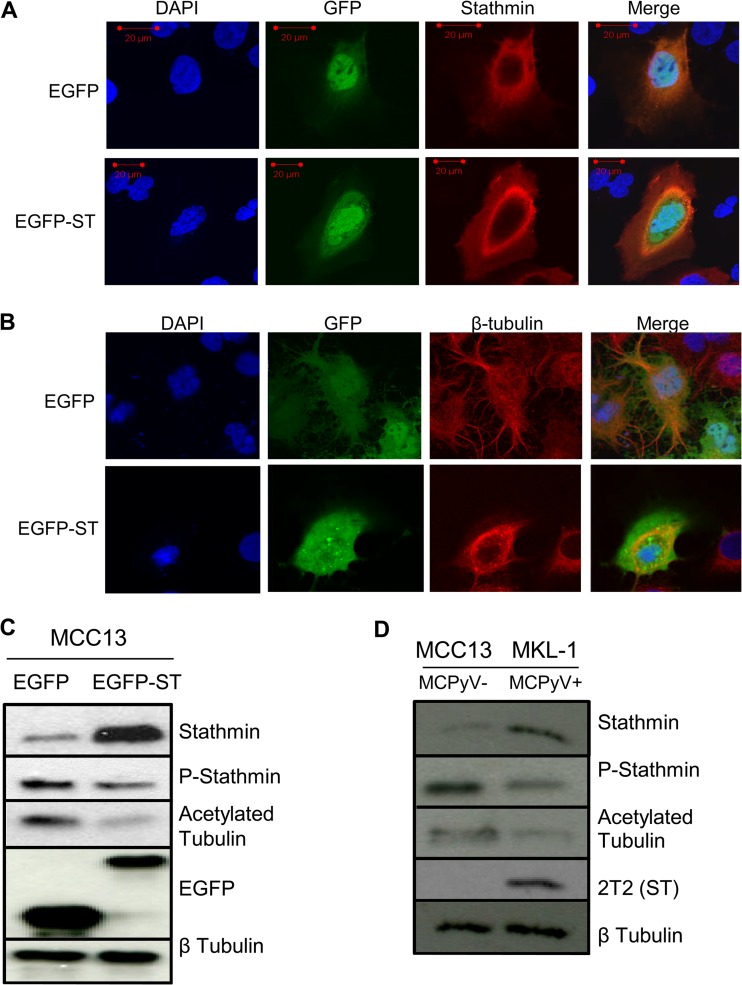
MCPyV ST promotes microtubule destabilization. MCC13 cells were transfected with either EGFP or EGFP-ST expression vectors. After 24 h, cells were fixed and permeabilized, and GFP fluorescence was analyzed by direct visualization, whereas endogenous stathmin (A) and endogenous β-tubulin (B) were identified by indirect immunofluorescence using stathmin- and β-tubulin-specific antibodies, respectively. (C) MCC13 cell lysates expressing EGFP or EGFP-ST were analyzed by immunoblotting using stathmin-, phosphorylated stathmin-, acetylated tubulin-, GFP-, and β-tubulin-specific antibodies. (D) Cellular lysates from MCC13 (MCPyV-negative) and MKL-1 (MCPyV-positive) cells were analyzed by immunoblotting using stathmin-, phosphorylated stathmin-, acetylated tubulin-, 2T2-, and β-tubulin-specific antibodies.

To investigate what effect MCPyV ST expression had upon stathmin subcellular localization, immunofluorescence studies were performed in MCC13 cells expressing EGFP or EGFP-ST, using a stathmin-specific antibody. The results show that endogenous stathmin has a diffuse cytoplasmic staining in EGFP-expressing cells. In contrast, upon MCPyV ST expression, stathmin is redistributed to a halo-like pattern around the nucleus, which colocalizes with a portion of MCPyV ST (Fig. 4A). This cytoplasmic halo-like staining is indicative of a destabilized microtubule network (31, 59). To determine if the stability of the microtubule network is altered upon MCPyV ST expression, immunofluorescence studies were repeated using a β-tubulin-specific antibody. In EGFP-transfected cells, β-tubulin highlighted the presence of a stable microtubule network, whereas in MCPyV ST-expressing cells, β-tubulin relocalized to a halo-like distribution, consistent with that of stathmin, indicating microtubule network destabilization (Fig. 4B).

To verify that the stability of the microtubule network is altered upon MCPyV ST expression, immunoblotting was performed using an acetylated tubulin-specific antibody, a marker of stabilized microtubules (60) in MCC13 cells transfected with either EGFP or EGFP-ST (Fig. 4C), or MCPyV-positive (MKL-1) versus the MCPyV-negative (MCC13) cells (Fig. 4D). Although no overall change in microtubule levels was observed, as indicated by β-tubulin levels, a marked decrease in acetylated tubulin levels was observed upon MCPyV ST expression, indicating a decrease in stabilized microtubules. Moreover, we also assessed whether MCPyV ST affects the phosphorylation status of stathmin, using phospho-stathmin (Ser16) and stathmin-specific antibodies. The results showed increased stathmin expression, as previously described (Fig. 4C and D). However, importantly, the levels of phosphorylated stathmin decreased upon MCPyV ST expression (Fig. 4C and D), suggesting that MCPyV ST leads to an increase in the pool of unphosphorylated stathmin, which in turn promotes microtubule destabilization.

### Microtubule destabilization is required for MCPyV ST-mediated cell motility.

**FIG 5:**
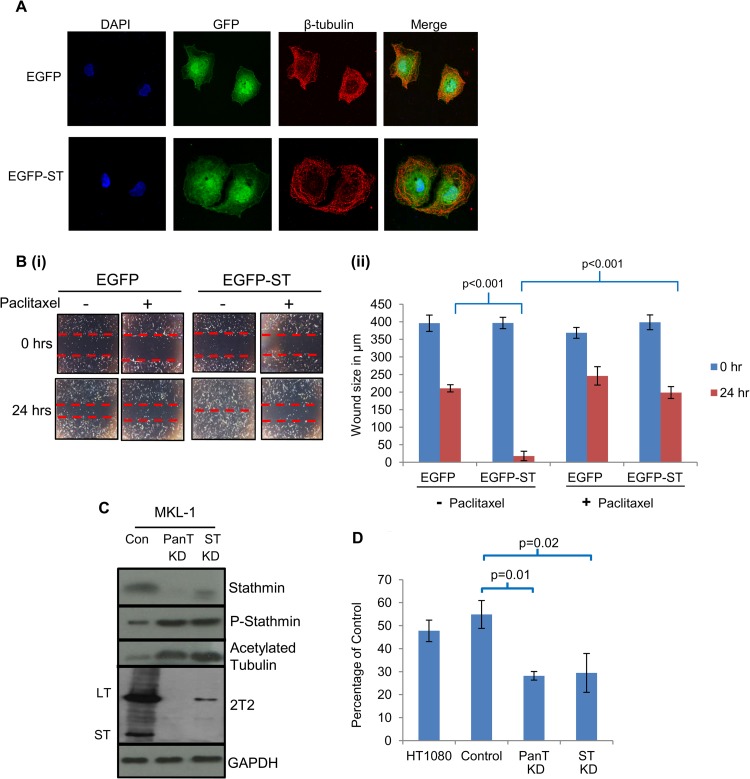
Microtubule destabilization is required for MCPyV ST-mediated cell motility. (A) MCC13 cells expressing EGFP or EGFP-ST were incubated in the presence of paclitaxel. After 24 h, cells were fixed and permeabilized, and GFP fluorescence was analyzed by direct visualization, whereas endogenous β-tubulin was identified by indirect immunofluorescence using a β-tubulin-specific antibody. (B) (i) MCC13 cells were transfected with either EGFP or EGFP-ST expression vectors and also incubated in the absence or presence of paclitaxel. After 24 h, a scratch was created by scraping the monolayer, migration of cells toward the scratch was observed over a 24-h period, and images were taken at 0 and 24 h under a Zeiss light microscope at ×4 magnification. (ii) The size of the wound was measured at 0 and 24 h. Scratch assays were performed in triplicate. (C) (i) MKL-1 cells were transduced with lentivirus-based vectors expressing shRNAs targeting ST alone, both LT and ST (PanT), and a scrambled negative control (Con). Cellular lysates were then analyzed by immunoblotting using stathmin-, phosphorylated stathmin-, acetylated tubulin-, and MCPyV T antigen (2T2)-specific antibodies. GAPDH was used as a measure of equal loading. (D) Precoated Matrigel-based Transwell migration tissue culture plates were seeded with MKL-1 cells transduced with scrambled (control)-, ST-, or ST-LT (PanT)-targeting lentivirus-based vectors. HT1080 cells, an invasive human fibrosarcoma cell line, were also used as a positive control. Cells were incubated in the Transwell plates for 22 h and then labeled with calcein AM fluorescent dye for 90 min, and fluorescence was measured. The experiment was performed in triplicate, and the graph indicates the fluorescence (percentage) of cells moving through the migration plates relative to the uncoated plate, set at 100%.

We next assessed if microtubule destabilization is essential for MCPyV ST-induced cell motility. The taxane-based drug paclitaxel binds microtubules, promoting polymerization and stability (61). Therefore, immunofluorescence studies were conducted to determine whether paclitaxel inhibited MCPyV ST-induced microtubule destabilization. Paclitaxel treatment had no effect on the stable microtubule network in EGFP-expressing MCC13 cells (Fig. 5A), resembling EGFP-expressing cells alone (Fig. 4B). However, paclitaxel inhibited EGFP-ST-mediated microtubule destabilization previously observed in Fig. 4B, since in the presence of the drug, distinctive bundles of microtubules were visible (Fig. 5A). To determine if prevention of MCPyV ST-induced microtubule destabilization affects cell motility and migration, scratch assays were performed comparing MCC13 cells expressing EGFP or EGFP-ST (Fig. 5B) in the absence and presence of paclitaxel. The results observed in the absence of paclitaxel were similar to those of Fig. 2B, demonstrating that MCPyV ST enhances cell motility and migration. In contrast, in the presence of paclitaxel, EGFP-ST-transfected MCC13 cells migrate at the same speed as EGFP-expressing MCC13 cells (Fig. 5B). These data suggest that MCPyV ST-mediated microtubule destabilization is required to enhance cell motility and migration, and the use of a microtubule-stabilizing drug can inhibit this function.

To confirm that MCPyV ST-induced microtubule destabilization is required for cell motility, depletion of ST was performed in the MCPyV-positive MKL-1 cell line using lentivirus-based vectors expressing shRNAs targeting ST alone, both LT and ST (“PanT” in Fig. 5B), and a scrambled negative control (10, 11). Results show that effective ST knockdown led to reduced levels of stathmin induction but increased levels of phosphorylated stathmin and acetylated tubulin, indicating increased microtubule stabilization compared to the scrambled control (Fig. 5C). This confirms that MCPyV ST is required for microtubule destabilization. Moreover, MKL-1 cells transduced with scrambled control-, ST-, or ST/LT-targeting lentivirus-based vectors were assessed for their ability to migrate in a Matrigel-based assay specific for nonadherent cells (62) (Fig. 5D). The results show that MCPyV ST depletion significantly reduced the motility of MKL-1 cells.

### Stathmin depletion inhibits MCPyV ST-mediated cell motility.

**FIG 6:**
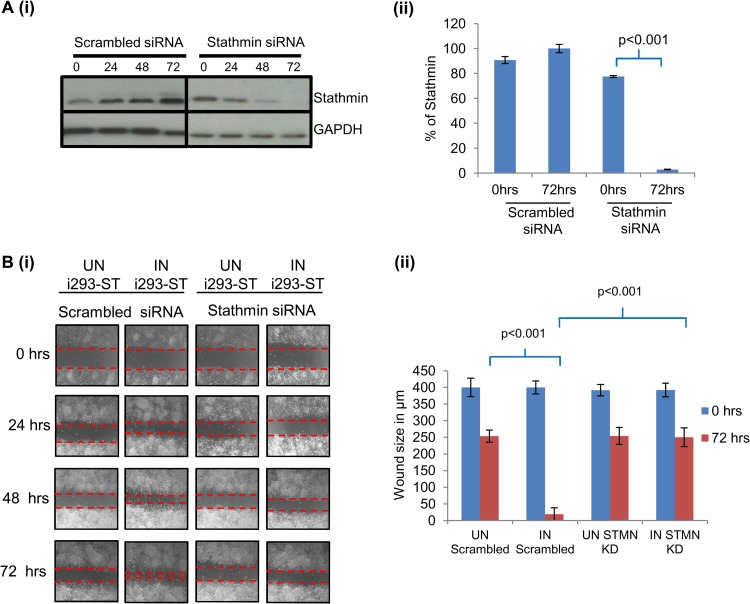
Stathmin is required for MCPyV ST-mediated cell motility. (A) (i) i293-ST cells were transfected with either scrambled siRNA or stathmin-specific siRNA and after 24 h remained uninduced or were induced with doxycycline hyclate. Cellular lysates were harvested every 24 h over a 72-h period, and immunoblotting was performed using stathmin- and GAPDH-specific antibodies (ii) Densitometry quantification of the Western blots was carried out using the ImageJ software and is shown as a percentage of relative densitometry of stathmin normalized to the loading control, GAPDH. (B) (i) Poly-l-lysine-coated 6-well plates were seeded with i293-ST cells and transfected with either scrambled siRNA or stathmin-specific siRNA, and after a further 12 h, cells either remained uninduced or were induced with doxycycline hyclate. After 24 h, a scratch was created by scraping the monolayer, migration of cells toward the scratch was observed over a 72-h period, and images were taken every 24 h under a Zeiss light microscope at ×4 magnification. (ii) The size of the wound was measured at 0 and 72 h. Scratch assays were performed in triplicate.

An important caveat to the paclitaxel experiment is that treatment of MCPyV ST-expressing cells with taxane-based drugs may also have an effect on cell division, preventing regrowth into the scratch. To overcome this and address the specific role of stathmin in MCPyV ST-mediated cell motility, scratch assays were performed comparing uninduced versus induced i293-ST cells, which were specifically depleted of stathmin (Fig. 6A). This experiment was performed with i293-ST cells due to the inability to effectively deplete stathmin levels in MKL-1 cells. i293-ST cells were initially transfected with either control scrambled or stathmin-specific siRNAs, and after 24 h, the cells remained uninduced or induced for MCPyV ST expression. Immunoblotting with a stathmin-specific antibody confirmed efficient knockdown (Fig. 6A). Similar results were observed in the presence of the scrambled siRNA control, as shown in Fig. 2B, where an increase in cell motility is observed upon MCPyV ST expression, resulting in a more rapid gap closure (Fig. 6B). In contrast, cellular growth back was inhibited in MCPyV ST-expressing i293-ST cells depleted for stathmin (Fig. 6B). Notably, these data demonstrate that stathmin is required for MCPyV ST-enhanced cell motility and migration.

### Cellular phosphatases are required for MCPyV ST-mediated microtubule destabilization and cell motility.

**FIG 7:**
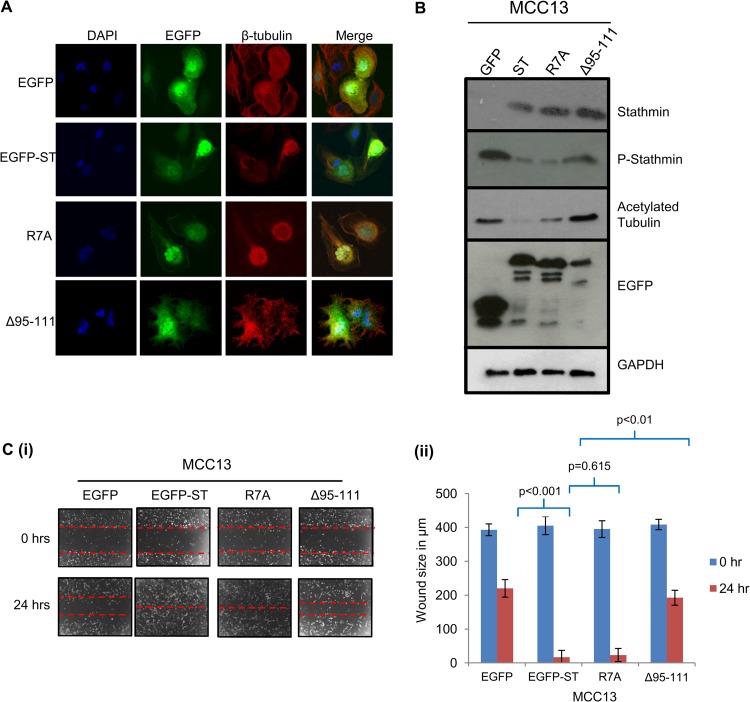
Cellular phosphatases are required for MCPyV ST-mediated microtubule destabilization and cell motility. (A) MCC13 cells were transfected with either EGFP, EGFP-ST, EGFP-R7A, or EGFP-Δ95-111 expression vector. After 24 h, cells were fixed and permeabilized, and GFP fluorescence was analyzed by direct visualization, whereas endogenous β-tubulin was identified by indirect immunofluorescence using a β-tubulin-specific antibody. (B) MCC13 cell lysates transfected with either EGFP, EGFP-ST, EGFP-R7A, or EGFP-Δ95-111 expression vector were analyzed by immunoblotting using stathmin-, phosphorylated stathmin-, acetylated tubulin-, and EGFP-specific antibodies. GAPDH was used as a measure of equal loading. (C) (i) MCC13 cells were transfected with either EGFP, EGFP-ST, EGFP-R7A, or EGFP-Δ95-111 expression vector. After 24 h, a scratch was created by scraping the monolayer, migration of cells toward the scratch was observed over a 24-h period, and images were taken at 0 and 24 h under a Zeiss light microscope at ×4 magnification. (ii) The size of the wound was measured at 0 and 24 h. Scratch assays were performed in triplicate.

The results suggest that MCPyV ST increases the pool of unphosphorylated stathmin levels. As MCPyV ST interacts with multiple cellular phosphatase subunits, including PP2A Aα, PP2A Aβ, and PP4C (10, 15), we assessed whether cellular phosphatases are required for microtubule destabilization and cell motility using two previously generated MCPyV ST mutants that fail to interact with PP2A Aα (the R7A mutant) or PP4C and PP2A Aβ (the Δ95-111 mutant) (15). Importantly, these mutants still retain other MCPyV ST functions (15). MCC13 cells expressing EGFP, EGFP-ST, EGFP-R7A, or EGFPΔ95-111 were stained with a β-tubulin-specific antibody to determine the presence of a stable microtubule network (Fig. 7A). R7A (ΔPP2A Aα) retained the ability to destabilize microtubules. Conversely, a stable microtubule network was evident in EGFPΔ95-111-expressing cells (ΔPP4C/PP2A Aβ). To confirm these observations, immunoblotting was performed with stathmin-, phosphorylated stathmin-, and acetylated tubulin-specific antibodies on MCC13 transfected cell lysates (Fig. 7B). Increased levels of phosphorylated stathmin and acetylated tubulin were observed in EGFPΔ95-111-expressing cells, compared to EGFP-ST- and EGFP-R7A-expressing cells, indicating increased microtubule stabilization. To test the requirement for cellular phosphatases in MCPyV ST-induced cell motility, scratch assays were performed with MCC13 cells (Fig. 7C) expressing EGFP, EGFP-ST, EGFP-R7A, or EGFPΔ95-111. The results demonstrate that cellular growth back was reduced in the EGFPΔ95-111-expressing compared to the EGFP-R7A- and MCPyV EGFP-ST-expressing cells. Together, the results suggest that PP4C and/or PP2A Aβ is required for MCPyV ST-induced cell motility and migration.

**FIG 8:**
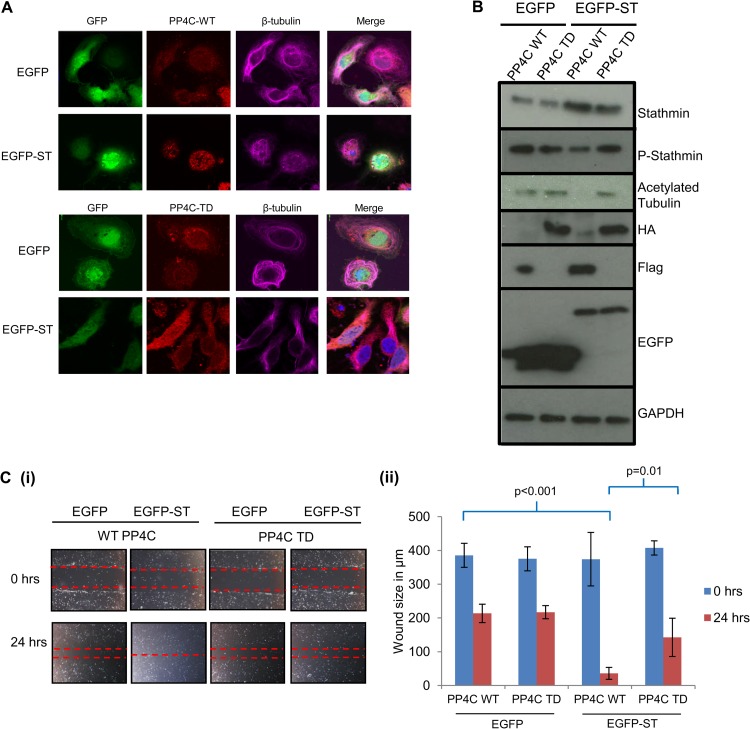
PP4C transdominant mutant expression inhibits MCPyV ST-mediated microtubule destabilization and cell motility. (A) MCC13 cells were transfected with either EGFP or EGFP-ST in the presence of WT PP4C or PP4C transdominant mutant expression vectors. After 24 h, cells were fixed and permeabilized, and GFP fluorescence was analyzed by direct visualization, whereas endogenous β-tubulin was identified by indirect immunofluorescence using a β-tubulin-specific antibody. (B) MCC13 cell lysates transfected with either EGFP or EGFP-ST in the presence of WT PP4C or the PP4C transdominant mutant were analyzed by immunoblotting using stathmin-, phosphorylated stathmin-, acetylated tubulin-, HA-, FLAG- and EGFP-specific antibodies. GAPDH was used as a measure of equal loading. (C) (i) MCC13 cells were transfected with either EGFP or EGFP-ST in the presence of WT PP4C or PP4C transdominant mutant expression vector. After 24 h, a scratch was created by scraping the monolayer, migration of cells toward the scratch was observed over a 24-h period, and images were taken at 0 and 24 h under a Zeiss light microscope at ×4 magnification. (ii) The size of the wound was measured at 0 and 24 h. Scratch assays were performed in triplicate.

Similar assays were also performed using wild-type or transdominant mutants of PP2A and PP4. PP2AcH118N, is a catalytically inactive transdominant mutant of the PP2A catalytic subunit (63), and PP4-RL is a phosphatase-dead mutant of PP4 (64). The presence of a stable microtubule network was analyzed using a β-tubulin-specific antibody in MCC13 cells expressing EGFP or EGFP-ST in the presence of either wild-type or catalytically inactive forms of PP2A or PP4 (Fig. 8A). The results show that expression of both wild-type cellular phosphatases had little effect on the ability of MCPyV ST to destabilize microtubules. However, upon expression of PP2AcH118N, destabilization of the microtubule network was evident in both control and MCPyV ST-expressing cells, suggesting that inhibition of PP2A activity induces microtubule destabilization (data not shown). This result has been previously observed (59, 65), and therefore no further conclusions on MCPyV ST function could be drawn using this mutant. In contrast, expression of PP4-RL had little effect on the microtubule network in control cells. Notably, however, a stable microtubule network was evident in MCPyV ST-expressing cells, suggesting that the PP4C transdominant mutant inhibited MCPyV ST-induced microtubule destabilization (Fig. 8A). To confirm these observations, immunoblotting was performed with stathmin-, phosphorylated stathmin-, and acetylated tubulin-specific antibodies on MCC13 cell lysates (Fig. 8B). Similar to EGFPΔ95-111-expressing cells, increased levels of phosphorylated stathmin and acetylated tubulin were observed in the presence of the PP4C transdominant mutant compared to the wild-type construct in MCPyV ST-expressing cells. We next assessed the effect of PP4-RL expression on MCPyV ST-induced cell motility using scratch assays, comparing MCC13 cells expressing EGFP or EGFP-ST in the presence of wild-type or the catalytically inactive PP4C (Fig. 8C). The results demonstrate that cellular growth back was inhibited in MCPyV ST-expressing cells transfected with the catalytically inactive PP4C, in contrast to wild-type PP4C. Together the results implicate PP4C in MCPyV ST-induced microtubule destabilization and cell motility.

## DISCUSSION

MCPyV ST is an oncogenic protein (10). Herein, we show that MCPyV ST leads to changes in cellular protein levels that regulate cytoskeletal organization and dynamics. Considering the highly metastatic nature of MCC, this suggests a possible link between MCPyV ST and cell motility and migration, essential factors for tumor cell dissemination. Indeed metastasis to distant sites correlates with poor MCC survival rates (16). MCC is effectively an accidental endpoint for MCPyV infection, due to the clonally integrated genome being unable to replicate. However, the promotion of metastasis by virus oncoproteins is not without precedent: a number of DNA viruses have been shown to induce metastasis by deregulating the normal processes of cellular adhesion and motility (66–69).

Stathmin is overexpressed in many cancer types (35, 36), but its contribution to tumor development is still poorly understood. The link between stathmin, microtubule destabilization, and cell motility is supported by the use of two stathmin mutants that separate its tubulin-sequestering activity, which destabilizes microtubules, from its catastrophe-promoting ability (35, 70). 3D Transwell migration and invasion assays overexpressing the wild type or the mutant containing the tubulin-sequestering activity dramatically decreased the amount of stable acetylated microtubules enhancing cell migration, in contrast to the catastrophe-promoting mutant (35). Interestingly, in contrast to 3D matrices or *in vivo* analysis, reports have suggested that in certain cell types, increased stathmin expression is insufficient to enhance cell motility in 2D time-lapse wound closure assays (35, 71, 72). This observation has yet to be fully elucidated but may be linked to distinct molecular mechanisms regulating cell morphology (73, 74). Expression of a less phosphorylated stathmin mutant can induce a rounded cell shape coupled with amoeboid-like protrusions, allowing enhanced movement using bleb-like propulsion mechanisms (35). It may be the case that stathmin overexpression leads to a more flexible and dynamic microtubule network, inducing a switch in mechanisms that further regulate additional cell morphology changes. Notably, bioinformatic analysis identified gene ontology headers that included general cytoskeletal changes within the MCPyV ST proteomic data, and work is ongoing to determine whether additional changes to the host cell actin cytoskeleton are induced upon MCPyV ST expression.

The results also suggest that the pool of unphosphorylated stathmin is increased upon MCPyV ST expression. To date, few cellular phosphatases have been implicated in the regulation of stathmin. Okadaic acid-mediated inhibition results in a major increase in the level of phosphorylation of stathmin (75), suggesting a possible role of protein phosphatases of types 1, 2A, and 2B. Moreover, *in vitro* dephosphorylation assays showed differential patterns of site-specific dephosphorylation, suggesting stathmin phosphorylation is modulated by the sequential activity of multiple cellular phosphatases (75). MCPyV ST interacts with various cellular phosphatase subunits, and we highlight a likely role of PP4C in MCPyV ST-induced microtubule destabilization and cell motility. Interestingly, PP4C has been implicated in microtubule dynamics and is required for proper microtubule organization at the centrosome through regulation of NDEL1 and recruitment of katanin p60 (76).

MCC is treated by surgical excision and radiotherapy, which can achieve loco-regional control, but a paucity of trials means no defined chemotherapy regime has proven effective for the metastatic disease. MCPyV may identify potential novel virus-targeted therapeutic regimens. For example, YM155, a small-molecule inhibitor of the survivin promoter, initiates cell death in virus-positive MCC cells and significantly delays MCC xenografts (77). MCPyV ST-induced microtubule destabilization may also be an avenue for further investigation. Taxanes are widely used in combination with other anticancer drugs to treat many different types of cancer and may be of therapeutic benefit for MCC, although the clinical utility of these microtubule inhibitors is often constrained by primary or acquired resistance. New microtubule-targeting agents that overcome taxane resistance provide additional options for treatment. The epothilones have activity against taxane-resistant cell lines and tumor xenografts (61). Therefore, it would be interesting to determine if epothilones also have inhibitory effects on MCPyV ST-induced cell migration. Moreover, the upregulation of stathmin by MCPyV ST may also be a novel therapeutic target. Gene profiling of melanoma cells has identified stathmin as a potential target of miR-193b, and ectopic expression of miR-193b results in a decrease in stathmin and reduced migration of multiple melanoma cell lines (38). As such, RNAi-based therapies delivering miR-193 mimics or stathmin-specific siRNAs may prove powerful tools to inhibit metastatic MCC.

In summary, we provide evidence that MCPyV ST promotes differential expression of cellular proteins implicated in cytoskeletal organization and dynamics leading to a motile and migratory phenotype. We demonstrate the essential role of stathmin in MCPyV ST-mediated microtubule destabilization and cell motility and implicate the cellular phosphatase catalytic subunit PP4C in the regulation of this process. These findings suggest a possible molecular explanation for the highly metastatic phenotype associated with MCCs and highlight stathmin as a possible biomarker of MCC prognosis and as a target for novel antitumoral therapies.

## Supplementary Material

Supplemental material
