# Supplementary material for: Merkel Cell Polyomavirus Small T Antigen Mediates Microtubule Destabilization To Promote Cell Motility and Migration
Source: J Virol. 2014 Dec 16;89(1):35–47. doi: 10.1128/JVI.02317-14 (PMC4301106; doi:10.1128/JVI.02317-14)
Supplement: Supplemental material [file supp_89_1_35__index.html]

Merkel Cell Polyomavirus Small T Antigen Mediates Microtubule Destabilization To Promote Cell Motility and Migration — Supplemental material 

# Merkel Cell Polyomavirus Small T Antigen Mediates Microtubule Destabilization To Promote Cell Motility and Migration

## Supplemental material

**Files in this Data Supplement:**

- Supplemental file 1 -

  Fig. S1 (MCPyV ST expression leads to differential expression of proteins involved in microtubule-associated cytoskeletal organization and dynamics.)

  Fig. S2 (Additional gene ontology groupings and pathway hits from SILAC analysis of i293-ST cells.)

  Legends to Movies S1 and S2

  PDF, 178K
- Supplemental file 2 -

  Movie S1 (293 cells transfected with an EGFP expression vector imaged after 12 hours with an Incucyte kinetic live cell imaging system every 30 minutes for 24 hours.)

  AVI, 7.9M
- Supplemental file 3 -

  Movie S2 (293 cells transfected with an EGFPST expression vector imaged after 12 hours with an Incucyte kinetic live cell imaging system every 30 minutes for 24 hours.)

  AVI, 8.6M
