# Supplementary material for: Merkel Cell Polyomavirus Small T Antigen Mediates Microtubule Destabilization To Promote Cell Motility and Migration
Source: J Virol. 2014 Dec 16;89(1):35–47. doi: 10.1128/JVI.02317-14 (PMC4301106; doi:10.1128/JVI.02317-14)
Supplement: Supplemental material [file JVI.02317-14_zjv999099857so1.pdf]

Supplementary Information

Merkel cell polyomavirus small T antigen mediates microtubule destabilisation to promote cell motility and migration

Laura M. Knight[1], Gabriele Stakaityte[1], Jennifer, J. Wood[1], Hussein Abdul-Sada[1], David A. Griffiths[1], Gareth J. Howell[1], Rachel Wheat[3], G. Eric Blair[1], Neil M. Steven[3], Andrew Macdonald[1,2], David J. Blackbourn[4] and Adrian Whitehouse[1,2,*]

[1]School of Molecular and Cellular Biology, [2]Astbury Centre for Structural Molecular Biology, University of Leeds, Leeds, LS2 9JT, [3]School of Cancer Sciences, University of Birmingham, Birmingham, B15 2TT and [4]School of Biosciences & Medicine, University of Surrey, Surrey, GU2 7XH United Kingdom.

*Correspondence to Adrian Whitehouse
Tel: +44 (0)113 3437096
Email: a.whitehouse@leeds.ac.uk

A

Gene Ontology headers
1. Cytoskeletal regulation, organisation and dynamics<br>2. Microtubule cytoskeletal organisation<br>3. Microtubule based movement and motility<br>4. Microtubule motor activity<br>5. Head and neck cancer<br>6. Regulation of Microtubule depolymerisation and polymerisation<br>7. Establishment and maintenance of cell polarity

B

Protein	IPI Accession no.	Function	Fold increase	Peptide hits
**Tubulin-specific chaperone A**	IPI00217236	Tubulin-folding protein	7.23	10
**Microtubule—associated protein, RP/EB family member 1**	IPI00017596	Regulates the microtubule dynamics by promoting nucleation and elongation	2.92	13
**Microtubule-associated protein 1B (MAP1B)**	IPI00008868	Involved in cell polarisation – required for efficient cross talk between microtubules and actin cytoskeleton	2.4	59
**Dystonin (DST)**	IPI00645369	Cytoskeletal linker protein – regulates keratinocyte polarity and motility	2.0	155
**Dynamin (DMN2)**	IPI00012837	Microtubule motor protein involved in distribution of mitochondria and lysosomes	2.56	40
**Stathmin 1**	IPI00479997	Involved in the regulation of the microtubule cytoskeletal network	8.33	11
**Microtubule-associated protein 4**	IPI00396171	Promotes microtubule assembly and disassembly	3.72	32
**Kinesin-like protein 14 (KIF14)**	IPI00299554	A microtubule motor protein essential for cell cytokinesis	11.39	2

Supplementary Figure 1. MCPyV ST expression leads to the differential expression of proteins involved in microtubule-associated cytoskeletal organisation and dynamics. (**a**) Bioinformatical analysis highlighted that a number of highly differentially expressed proteins upon MCPyV ST expression were implicated in gene ontology groupings involving microtubule-associated cytoskeletal organisation and dynamics. (**b**) Summary of microtubule-associated regulatory proteins identified in i293-ST SILAC-based quantitiative proteomic screen.

Additional Cytoskeletal Regulation, Organisation and Dynamics, Cell Morphology, Establishment of Localisation and directed movement

Uniprot Number	Protein	Fold Increase	Number of Peptide Hits
P33176	KIF5B	2.6	40
O75369	FLNB	2.6	105
P07355	ANXA2	3.5	20
P46939	UTRN	2.0	49
Q9ULV4	CORO1C	4.0	13
Q14247	CTTN	3.7	17
P61160	ARP2	3.3	11
P61158	ARP3	2.1	13
P61586	RHOA	3.7	9
P07737	PFN1	6.7	10
P60981	DSTN	5.0	6
P23528	CFL1	5.0	16
P18206	VCL	5.3	42
P13797	PLS3	5.6	28
P29966	MARCKS	6.2	7

DNA Replication, recombination and repair, Cell cycle regulation, proliferation and growth

Uniprot Number	Protein	Fold Increase	Number of Peptide Hits
P60953	CDC42	7.00	5
P04114	APOB	41.0	4
Q9NVV4	MTPAP	2.0	5
P28340	POLD1	2.0	5
P00441	SOD1	8.6	8
Q9NRR5	UBQLN4	3.0	7
P20248	CCNA2	1.9	2
P24941	CDK2	2.0	7
Q9Y5K6	CD2AP	2.7	9
P62826	RAN	7.0	7
Q96GD4	AURKB	1.6	6
P28482	MAPK1	2.9	12
Q9Y3F4	STRAP	3.6	17

Post-translational modification, protein turnover, chaperones, Intracellular trafficking and secretion

Uniprot Number	Protein	Fold Increase	Number of Peptide Hits
O43301	HSPA12A	2.1	3
O14773	TPP1	2	2
O95487	SEC24B	2	2
D4A2D7	Ipo4	2	24
O00505	KPNA3	2	10
Q15833	STXBP2	2.6	3
Q5THJ4	VPS13D	2	6
Q9BTE6	AARSD1	2.1	2
Q5T160	RARS2	2	2
P41250	GARS	2.1	24
O00303	EIF3F	4.6	8

Supplementary Figure 2. Additional gene ontology groupings and pathway hits from SILAC analysis of i293-ST cells. Further bioinformatical analysis highlighting a number of highly differentially expressed proteins upon MCPyV ST expression and their respective gene ontology groupings.

Supplementary Movies. 293 cells were transfected with EGFP (**Movie 1**) and EGFP-ST (**Movie 2**) expression vectors. After 12 hours, the cells were images using an Incucyte kinetic live cell imaging system. Imaging was performed for a 24 hour period, with images taken every 30 minutes.
